# Role of acid sphingomyelinase bioactivity in human CD4^+^ T-cell activation and immune responses

**DOI:** 10.1038/cddis.2015.178

**Published:** 2015-07-23

**Authors:** A Bai, E Kokkotou, Y Zheng, S C Robson

**Affiliations:** 1 Division of Gastroenterology, Department of Medicine, Beth Israel Deaconess Medical Center, Harvard University, Boston, MA, USA; 2 Department of Surgery, Beth Israel Deaconess Medical Center, Harvard University, Boston, MA, USA

## Abstract

Acid sphingomyelinase (ASM), a lipid hydrolase enzyme, has the potential to modulate various cellular activation responses via the generation of ceramide and by interaction with cellular receptors. We have hypothesized that ASM modulates CD4^+^ T-cell receptor activation and impacts immune responses. We first observed interactions of ASM with the intracellular domains of both CD3 and CD28. ASM further mediates T-cell proliferation after anti-CD3/CD28 antibody stimulation and alters CD4^+^ T-cell activation signals by generating ceramide. We noted that various pharmacological inhibitors of ASM or knockdown of ASM using small hairpin RNA inhibit CD3/CD28-mediated CD4^+^ T-cell proliferation and activation. Furthermore, such blockade of ASM bioactivity by biochemical inhibitors and/or molecular-targeted knockdown of ASM broadly abrogate T-helper cell responses. In conclusion, we detail immune, pivotal roles of ASM in adaptive immune T-cell responses, and propose that these pathways might provide novel targets for the therapy of autoimmune and inflammatory diseases.

Acid sphingomyelinase (ASM), a lipid hydrolase enzyme localized to lysosomes and cell membranes, converts sphingomyelin to ceramide,^1^ an important lipid messenger mediating cell signaling.^2, 3^ Through the generation of ceramide, ASM appears to have an important role in regulating cell differentiation, proliferation, and apoptosis.^1, 4^ Abnormalities in ASM bioactivity result in multiple system disorders. As an example, patients with Niemann–Pick disease, who have mutations in the *ASM* gene, exhibit neurological symptoms at early age, and develop visceral organ abnormalities in later life.^4^ Patients with Niemann–Pick disease are at risk of infections,^5^ as can be modeled in ASM-deficient mice.^6, 7^ This phenotype has been attributed to phagocyte dysfunction.^8^ Recently, however, ASM function has also been described and noted in various other non-phagocytic immune cells, for example, regulating cytotoxic granule secretion by CD8^+^ T cells.^9^

ASM has been reported to modulate T-cell receptor (TCR) signaling initiated by TNF,^10^ mediate CD28 signals,^11^ and induce or rescue CD4^+^ T cells from apoptosis under certain circumstances.^12, 13^ By generating ceramide, ASM serves as a regulator of intracellular downstream signaling. However, the exact manner whereby ASM participates in TCR/CD3 or/and CD28 signaling remains controversial.^10, 11, 14^ Furthermore, the molecular mechanisms as to how ASM regulates CD4^+^ T-cell activation are still largely unexplored.

Adaptive immune responses are important in the maintenance of human immune homeostasis. Imbalances in T-helper cell (Th) responses associated with aberrant CD4^+^ T-cell activation contribute to the development of inflammation as in human autoimmune diseases.^15, 16^ It remains unclear whether or how ASM might dictate Th responses during the progression of inflammatory diseases.

In the present study, we confirm that ASM interacts with CD3 and CD28, and mediates intracellular signals that control CD4^+^ T-cell activation. ASM inhibition either by pharmacological inhibitors of ASM or knockdown of ASM results in decreased ceramide production. This leads to non-responsiveness of CD4^+^ T-cell to CD3/CD28 engagement, and causes globally diminished Th responses. These data suggest the pivotal role of ASM in CD3/CD28 intracellular signaling and adaptive immune responses, and also provide a potential target for the therapy of immune disease.

## Results

### Treatment with ASM inhibitors abrogates naive CD4^+^ T-cell responses

**Figure 1:**
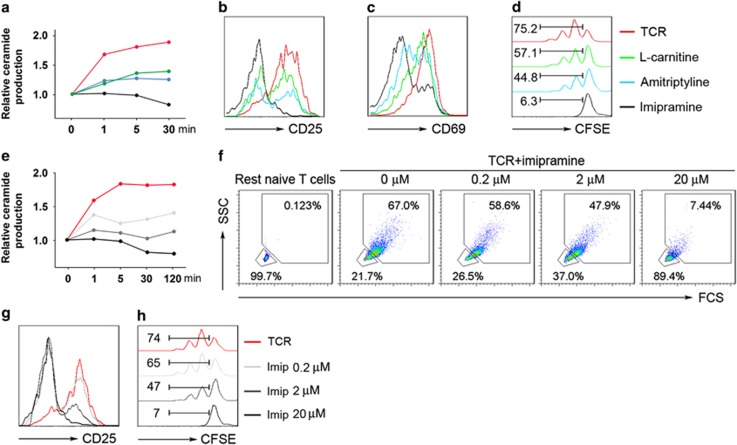
Heightened ASM activity during naive CD4^+^ T-cell activation and proliferation. (**a**–**d**) Naive CD4^+^ T cells (CD4^+^CD45RA^+^) were stimulated with anti-CD3/CD28 antibody-coated beads in the presence of l-carnitine (1 mM), amitriptyline (5 *μ*M), or imipramine (20 *μ*M) for different times as indicated, ceramide production was measured by TLC (**a**), expression of CD25 (**b**) and CD69 (**c**) at 48 h, or CFSE (**d**) at 72 h were determined by FACS (fluorescence-activated cell sorting ). (**e**–**h**) Anti-CD3/CD28 antibody-coated beads activated naive CD4^+^ T cells were treated with various concentrations of imipramine (0.2, 2, and 20 *μ*M) for different times, followed by TLC measurement of ceramide production (**e**), and FACS analysis of T-cell activation (**f**) and CD25 at 48 h (**g**), or CFSE (**h**) expression at 72 h. Data are representative of three to six independent experiments

Three specific ASM inhibitors inclusive of amitriptyline, l-carnitine, and imipramine were used to block ASM bioactivity in naive CD4^+^ T cells (CD4^+^CD45RA^+^) purified from healthy volunteer blood. Stimulation with anti-CD3/CD28 antibody-coated beads markedly induced ASM bioactivity as indicated by ceramide production determined by thin-layer chromatography (TLC), and this was significantly dampened by any of the three ASM inhibitors, with imipramine exhibiting the highest potency (Figure 1a). Comparable inhibitory effects of ASM inhibitors were observed on CD4^+^ T-cell activation (as marked by CD25 and CD69 expression ^17^) and proliferation as programmed by CD3 and CD28 dual engagement (Figures 1b–d).

Next, we further evaluated the phenotypic and functional impacts of imipramine. Imipramine substantially suppressed CD3/CD28 stimulation-induced ceramide generation, CD4^+^ T-cell activation characterized by increases in cell contents and size,^18^ and CD25 expression, as well as proliferation of naive CD4^+^ T cells, in a dose-dependent manner (Figures 1e–h).

### ASM inhibition by imipramine blocks CD3/CD28 signaling cascades in naive CD4^+^ T cell

**Figure 2:**
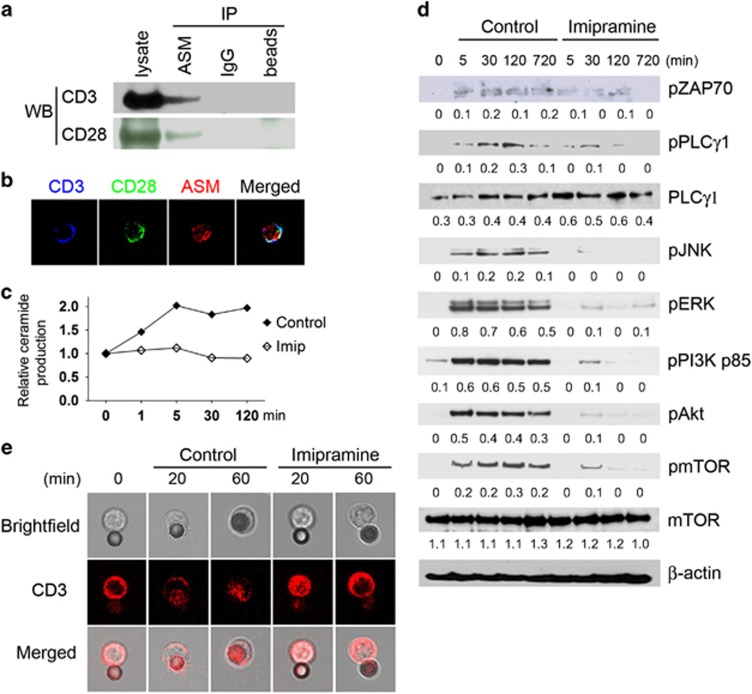
Association of ASM and CD3/CD28 in CD4^+^ T cells. (**a**) Co-immunoprecipitation of ASM with CD3 and CD28, respectively, from naive CD4^+^ T-cell lysates. Mouse IgG or beads only served as capture controls. (**b**) Colocalization of CD3/CD28 with ASM on naive CD4^+^ T cells was determined using confocal microscopy. (**c**–**e**) Naive CD4^+^ T cells were activated with anti-CD3/CD28 antibody-coated beads in the absence or presence of imipramine (20 *μ*M) for different times, ceramide production, intracellular CD3/CD28 signal transduction, and CD3 dynamics were examined by TLC (**c**), western blot using specific antibodies as indicated (**d**), and confocal microscopy (**e**), respectively. The beads adjacent to cells show reflected fluorescence. Data are representative of three to five independent experiments

Given these observations, we hypothesized that ASM acts as a key cell membrane protein-mediating intracellular signal cascades of CD3 and CD28. Then, we considered possible physical associations between ASM and CD3/CD28 molecules in naive CD4^+^ T cells. Both co-immunoprecipitation study and confocal microscopy have shown that ASM interacts with CD3 and CD28 in these cells (Figures 2a and b).

We have determined the intracellular signals of CD3/CD28 mediated by ASM. T-cell activation induced by anti-CD3/CD28 antibodies is characterized by two major signaling pathways: one including CD3, ZAP70, PLC-*γ*1, MAPK/JNK (CD3, ZAP70-phospholipase C-*γ*1, mitogen-activated protein kinase/c-Jun N-terminal kinase)^19, 20^ and the other CD28, PI3K, Akt, mTOR (CD28, phosphoinositide 3-kinase, Akt, mammalian target of rapamycin).^21, 22^ CD3/CD28 ligation also triggered increased ASM bioactivity (Figure 2c), activation of intracellular signal cascades of CD3/CD28 including ZAP70, PLC-*γ*1, JNK, ERK, PI3K, Akt, and mTOR (Figure 2d), and CD3 dynamics as shown by CD3 polarization toward the anti-CD3/CD28 antibody-coated beads (Figure 2e).^23^ The events above were specifically and substantially inhibited by blockade of ASM bioactivity with imipramine (Figures 2c–e).

**Figure 3:**
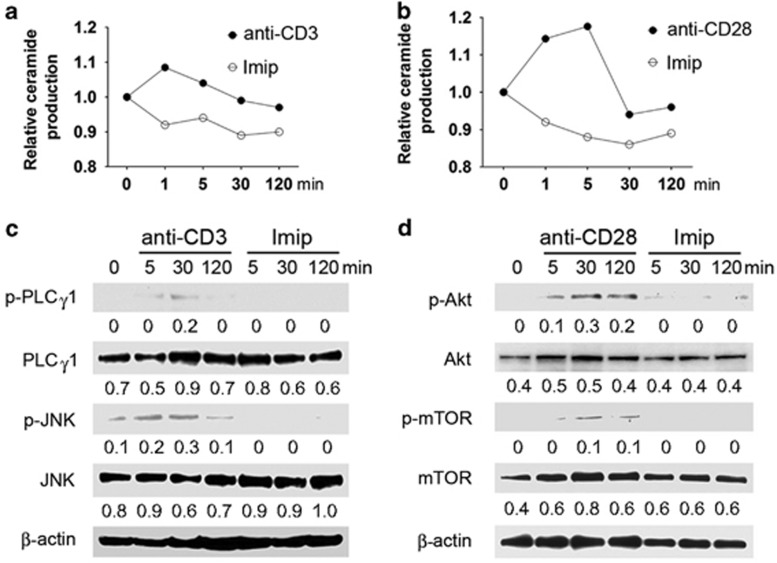
Imipramine inhibits CD3 or CD28 signaling through blockade of ASM bioactivity. Naive CD4^+^ T cells were stimulated with crosslinked antibodies to CD3 (10 *μ*g/ml) (**a** and **c**) or CD28 (10 *μ*g/ml) (**b** and **d**) in the absence or presence of imipramine (20 *μ*M) for different times, ceramide production and intracellular CD3 or CD28 signal cascades were measured by TLC (**a** and **c**) and western blot (**c** and **d**), respectively. Data are representative of three independent experiments

We then explored the role of ASM in transmitting individual signals of CD3 and CD28. Upon stimulation with crosslinked antibody to CD3 or CD28, ceramide production by naive CD4^+^ T cells reached high levels (at 1–5 min poststimulation), and returned to basal levels after 30 min (Figures 3a and b). In contrast, imipramine markedly dampened ceramide production (Figures 3a and b), concomitant with the blockade of intracellular signal cascades of either CD3 or CD28 (Figures 3c and d). These data indicate that ASM has pivotal roles in mediating CD3 and CD28 signal cascades and also in regulating CD4^+^ T-cell activation.

### Impacts of exogenous ceramide on naive CD4^+^ T-cell responses to CD3/CD28 engagement

**Figure 4:**
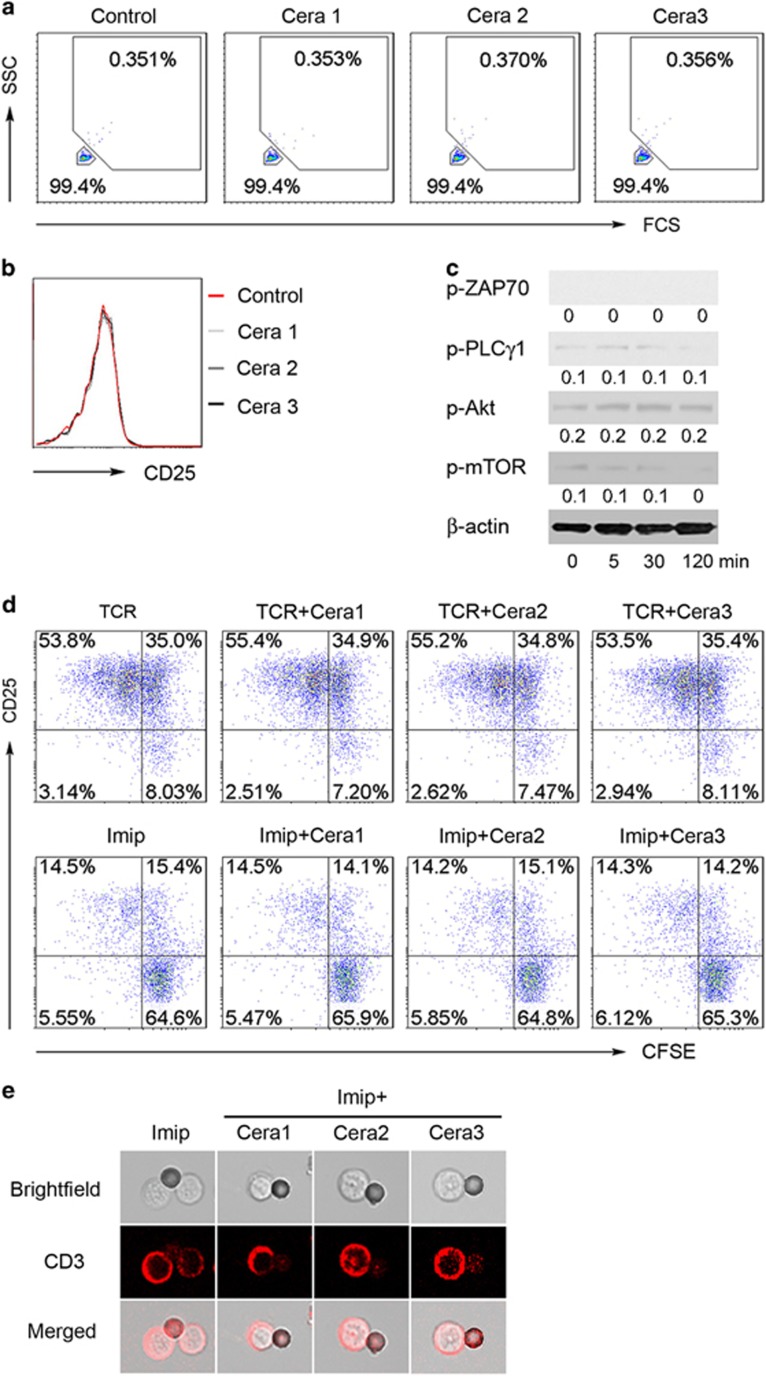
Exogenous ceramide fails to rescue CD4^+^ T cells from ASM inhibition. (**a**–**c**) Naive CD4^+^ T cells were stimulated with anti-CD3/CD28 and treated with various concentrations of exogenous C6-ceramide (Cera 1: 10 nM; Cera 2: 100 nM; Cera 3: 1000 nM), followed by FACS (fluorescence-activated cell sorting) analysis of T-cell activation (**a**) and CD25 expression (**b**) at 48 h, and western blotting of intracellular CD3/CD28 signaling components induced by 1000 nM C6-ceramide at the indicated time (**c**). (**d**) CFSE-labeled naive CD4^+^ T cells were stimulated with anti-CD3/28 antibody-coated beads for 72 h in the presence of different doses of C6-ceramide as indicated above together with or without imipramine (10 *μ*M), followed by FACS detection of CFSE and CD25 expression. (**e**) Anti-CD3/CD28 antibody-coated beads of activated CD4^+^ T cells were treated with imipramine (10 *μ*M) in combination with various doses of C6-ceramide as above, and CD3 dynamics was visualized at 60 min after anti-CD3/CD28 stimulation by confocal microscopy. Data are representative of three to five independent experiments

As ASM bioactivity/ceramide generation conveys CD4^+^ T-cell signaling and regulates cell activation, we next explored whether supplement of exogenous ceramide might induce CD4^+^ T-cell activation and therefore rescue CD4^+^ T cell from ASM inhibition. C6-ceramide has been reported as a permeable ceramide analog to trigger intracellular signaling of CD161 in human NK cells.^24^ To study the effect of exogenous ceramide, we placed C6-ceramide into the culture medium of CD4^+^ T cells. As shown in Figures 4a–c, treatment with different doses of C6-ceramide failed to induce extension of cell contents and size (Figure 4a), alter CD25 expression (Figure 4b), and had no effect on activation of intracellular signal cascades inclusive of PLC*γ*1, Akt, and mTOR in quiescent naive CD4^+^ T cells (Figure 4c). Note that the exogenous C6-ceramide is a permeable analog, and would perturb the ASM assay. From these derived data and others, we conclude that exogenous ceramide alone, the product of ASM, was not able to rescue CD4^+^ T-cell signaling from the impact of ASM enzymatic inhibition. Moreover, different doses of C6-ceramide did not alter characteristics of naive CD4^+^ T-cell activation and proliferation under CD3/CD28 dual engagement (Figure 4d).

To determine whether exogenous C6-ceramide might, however, rescue CD4^+^ T-cell activation from ASM inhibition, we stimulated naive CD4^+^ T cells with anti-CD3/CD28 antibody-coated beads in the presence or absence of C6-ceramide. Indeed, exogenous C6-ceramide had no effect on CD25 and CFSE (carboxyfluorescein diacetate succinimidyl ester) expression (Figure 4d) and on the typical CD3 dynamics (Figure 4e) in imipramine-treated CD4^+^ T cells. These data indicate that physical ASM bioactivity/ceramide production mediates CD4^+^ T-cell activation, and suggest that exogenous ceramide supplementation has no role in the regulation of CD4^+^ T-cell responses.

### Imipramine inhibits memory CD4^+^ T-cell activation

**Figure 5:**
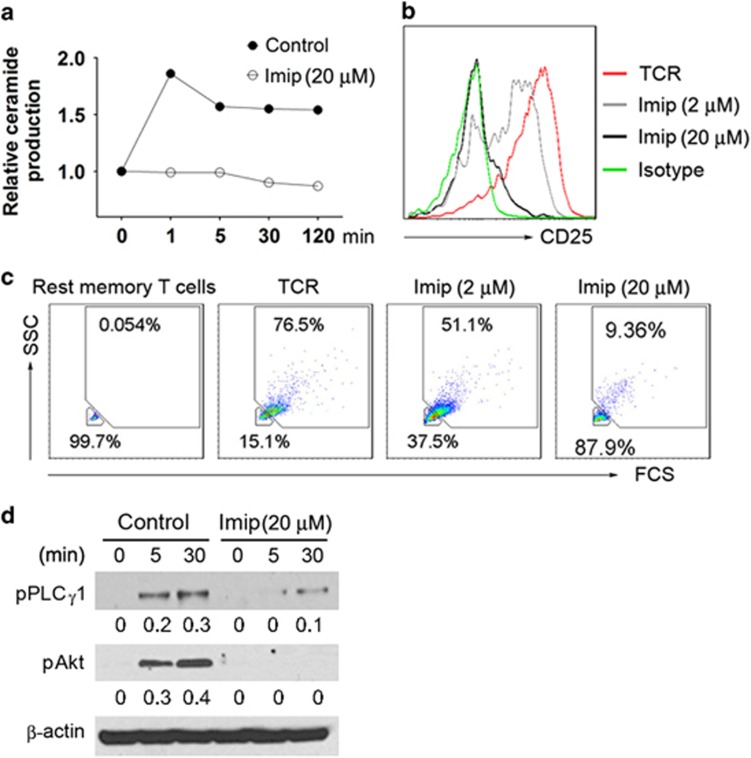
Imipramine inhibits memory CD4^+^ T-cell activation. CD4^+^ memory T cells (CD4^+^CD45RO^+^) were activated with anti-CD3/CD28 antibody-coated beads in the absence or presence of imipramine, followed by TLC measurement of ceramide production (**a**), FACS (fluorescence-activated cell sorting) analysis of CD25 expression (**b**) and T-cell activation (**c**) at 48 h, and western blot evaluation of intracellular CD3/CD28 signal components for different times (**d**). Data are representative of three independent experiments

Naive and memory CD4^+^ T cells have variant responsiveness to CD3 and CD28 engagement and exhibit diverse cell signaling in response to CD3 and CD28 stimulation, respectively.^25, 26^ We next evaluated sensitivity of memory CD4^+^ T cell (CD4^+^CD45RO^+^) to ASM inhibition. As shown in Figures 5a–d, substantial ASM bioactivity/ceramide production (Figure 5a), cell activation (Figures 5b and c), and intracellular signaling transduction, inclusive of PLC-*γ*1 and Akt (Figure 5d), were observed in memory CD4^+^ T cells after CD3/CD28 engagement. Moreover, ASM inhibition with imipramine diminished these changes of ASM bioactivity/ceramide production, cell activation, and intracellular signal transduction in memory CD4^+^ T cells (Figures 5a–d). This effect was concurrent with the inhibitory effect observed on naive CD4^+^ T cells.

### ASM inhibition blocks Th responses

**Figure 6:**
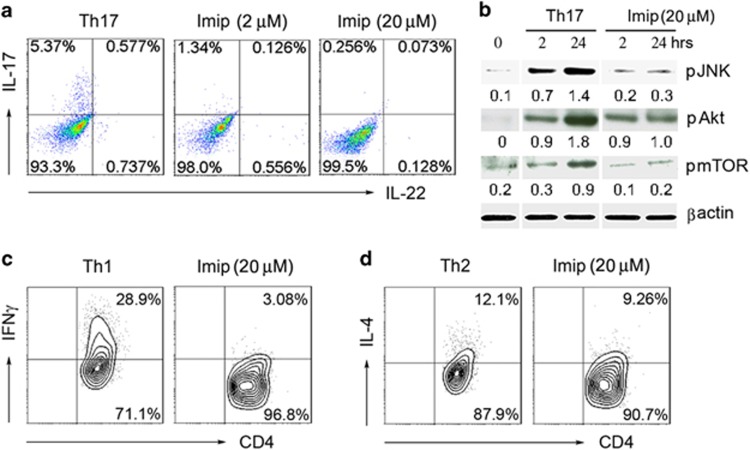
Imipramine is a global inhibitor of Th responses. (**a** and **b**) Naive CD4^+^ T cells were differentiated under Th17 condition in the absence or presence of imipramine, intracellular expression of interleukin-17 (IL-17) and IL-22, and Th17 relevant signals were determined by FACS (fluorescence-activated cell sorting ) analysis at 72 h (**a**) and western blotting measurement at 48 h (**b**), respectively. (**c** and **d**) Naive CD4^+^ T cells were differentiated into Th1 or Th2 cells in the absence or presence of imipramine (20 *μ*M) for 72 h, and intracellular levels of interferon-*γ* (IFN*γ*) (**c**) or IL-4 (**d**) were detected by FACS. Data are representative of three to four independent experiments

TCR/CD3 signal cascades in integration with costimulatory receptors such as CD28 signals are requisite for T-cell activation, and also for induction of Th differentiation, for example, Th1, Th2, and Th17.^27, 28^ We hypothesized that ASM could act to drive distinct Th cell differentiation. We first differentiated naive CD4^+^ T cells (CD4^+^CD45RA^+^) into Th17 cells, in the presence of imipramine. As shown in Figure 6a, imipramine downregulated IL-17 expression in CD4^+^ T cells under Th17-polarizing conditions. We further determined downstream cascades of CD3/CD28, and noted that ASM inhibition by imipramine blocked signaling cascades inclusive of Akt, mTOR, and JNK (Figure 6b). In parallel, imipramine inhibited IFN*γ* or IL-4 expression by CD4^+^ T cells under Th1 or Th2 deviating condition, respectively (Figures 6c and d). The data indicate the pivotal role of ASM bioactivity in the regulation of Th differentiation.

### Knockdown of ASM dampens CD3/CD28 signals and broadly inhibits Th responses

The results above indicate the pivotal role of ASM in T-cell activation and Th differentiation. To provide further direct evidence of associations between ASM bioactivity and T-cell function, we next used small hairpin RNA (shRNA) technology to knockdown ASM in healthy blood CD4^+^ T cells.

**Figure 7:**
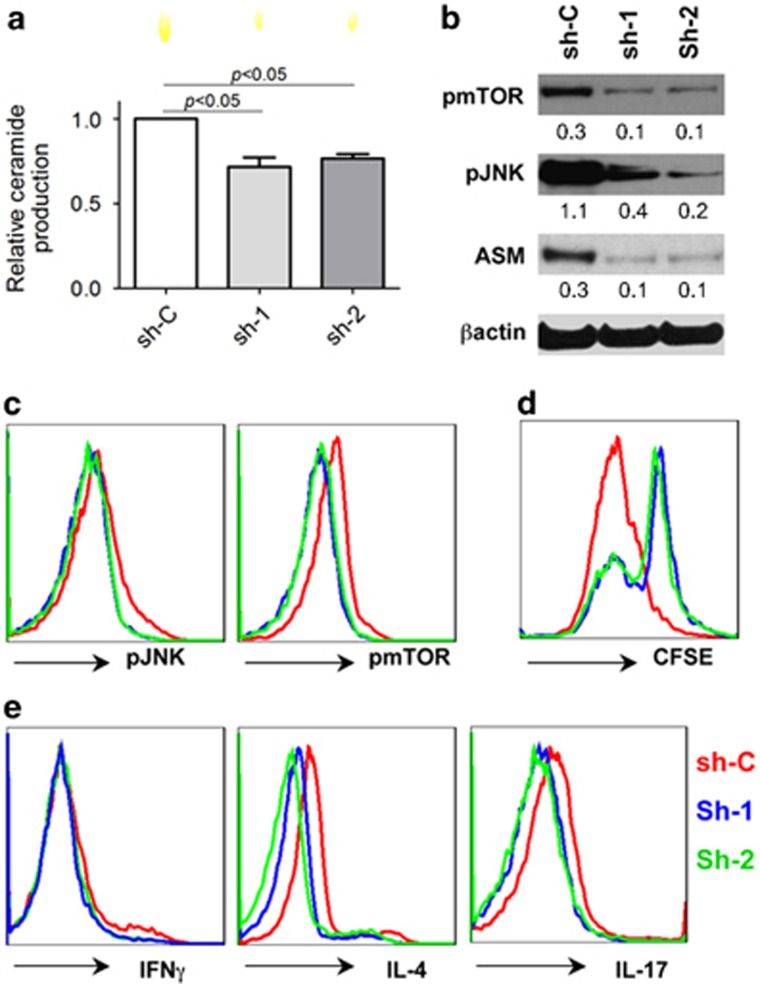
Knockdown of ASM blocks CD3/CD28 signals and Th responses. (**a**–**d**) Control knockdown (sh-C) and ASM knockdown (sh-1 and sh-2) of healthy blood CD4^+^ T cells were stimulated with anti-CD3/CD28 antibody-coated beads, followed by representative TLC analyses of ceramide at 120 min (**a**), CD3/CD28 signaling at 120 min by western blotting (**b**), and FACS (fluorescence-activated cell sorting ) analysis (**c**), and CFSE expression at 96 h (**d**). (**e**) ASM knockdown of CD4^+^ T cells above were cultured under Th1-, Th2-, and Th17-polarizing conditions, and the intracellular levels of IFN*γ*, IL-4, and IL-17 were determined by FACS at 96 h, respectively. Data are representative of three independent experiments

We used two efficient shRNA sequences in the study, and noted that knockdown of ASM by shRNA inhibited ASM bioactivity/ceramide production (Figure 7a). This change occurred concurrently with the blockade of CD3/CD28 signal components inclusive of mTOR and JNK (Figures 7b and c) and suppressed proliferation of CD4^+^ T cells (Figure 7d). Downregulation of ASM in CD4^+^ T cells markedly dampened Th1, Th2, and Th17 responses, respectively (Figure 7e). These data indicated ASM-dependent CD3/CD28 signal cascades and Th responses.

## Discussion

Sphingolipids, ceramide and ASM, have established effects in cellular signaling and in a variety of biological functions. These lipid pathways have also been implicated in human disorders including cancer, sepsis, and neurological disorders.^1^ Deficiency in ASM bioactivity results in impaired capability of macrophages to eradicate pathogens and release cytokines, as shown in experimental models and with ASM inhibitors.^8, 29^ Impaired innate immune function is also observed in Niemann–Pick disease patients with mutations in the *ASM* gene, resulting in heightened risk of patients to infections.^5^ The effect of ASM in adaptive immune responses remains largely unexplored, and the present study provides interesting evidence that ASM has a pivotal role in regulating CD4^+^ T-cell activation and function.

Boucher *et al.*^11^ have determined ceramide production in starved Jurkat cell lines by TLC after crosslinked anti-CD3 or anti-CD28 antibody (both 2 *μ*g/ml) stimulation. They noted that CD28 engagement induced a higher level of ceramide production, whereas CD3 stimulation triggered minimal ceramide generation, suggesting an important role of ASM in CD28 signaling.^11^ Others have noted that combinations of anti-CD3 (10 *μ*g/ml) and anti-CD28 (1 *μ*g/ml) antibodies induce IL-2 production in ASM deficiency splenocytes, and concluded that both CD3 and CD28 signal transductions were independent of ASM bioactivity and ceramide generation.^14^ However, another group reported that ASM was involved in modulating CD3/CD28 signaling in human CD4^+^ T cells.^10^

To clarify this area and to determine the role of ASM bioactivity in CD3 and CD28 signaling, we performed the present study, and noted that single crosslinked anti-CD3 or anti-CD28 antibody (both 10 *μ*g/ml) could exactly induce ASM bioactivity and ceramide production of CD4^+^ T cells. Following immunoprecipitation, we confirmed physical interaction of ASM with both CD3 and CD28, respectively. Hence, these studies support, at least in part, the earlier work by Boucher *et al.*,^11^ and indicate the potential for association of plasma membrane ASM with T-cell proteins CD3 and CD28.

Next, we determined CD3 and CD28 signals in CD4^+^ T cells. Single or dual CD3 and CD28 stimulation induced ceramide production, and gradually initiated intracellular signal cascades. In contrast, ASM pharmacological inhibition or knockdown of ASM blocked the signaling cascades of single or dual CD3/CD28 engagement. These data linked ASM bioactivity and ceramide to mediators of CD4^+^ T-cell signals and activation. Moreover, upon anti-CD3/CD28 antibody-coated bead stimulation, TCR/CD3 polarizes toward interacting areas, which enhances cell signaling.^23^ ASM inhibition by imipramine suppresses the polarization of TCR/CD3 repositioning induced by anti-CD3/CD28 engagements, which indicates that ASM not only mediates CD3 and CD28 signals but is also closely associated with activation-induced CD4^+^ T-cell polarization.

As one of the key lipid molecules, ceramide mediates cellular signals to regulate cell differentiation, proliferation, and apoptosis.^2, 3^ It has been reported that exogenous ceramide exhibits multiple functions in a dose-dependent manner. Treatment with high concentration (>10 *μ*M) of ceramide induces cell apoptosis^30^ and blocks cell signaling,^31^ whereas low titration (<1 *μ*M) of ceramide initiates intracellular PKB (Akt) activation.^24^ However, recent studies have shown different findings that exogenous ceramide displays no effect by its single treatment.^32^ Our data have shown that endogenous ceramide as a central messenger mediates CD3 and CD28 signal cascades, and responds to CD4^+^ T-cell activation. We noted that treatment with exogenous C6-ceramide, a permeable ceramide analog, had minimal effect on intracellular CD3/CD28 signals and CD4^+^ T-cell activation. Meanwhile, C6-ceramide failed to rescue T-cell activation from ASM inhibition. In the present study, we show the pivotal role of endogenous ceramide but not exogenous ceramide in mediating CD4^+^ T-cell signals and cell activation.

Autoimmune diseases such as arthritis and inflammatory bowel disease are characterized by CD4^+^ T-cell overactivation with excessive Th1 and Th17 responses.^33, 34, 35^ TCR/CD3 signaling cascades in combination with costimulatory CD28 signal induce T-cell activation, cell differentiation and cytokine expression, while dictating the progression of inflammation.^28^ We speculated that ASM bioactivity could also mediate CD4^+^ T-cell differentiation and Th cell responses. In this study, we demonstrate that ASM inhibition by imipramine or knockdown of ASM suppresses T-cell differentiation into Th1, Th2, and Th17 *in vitro*, through blockade of CD3/CD28 downstream signal cascades. The data provide the evidence that ASM activity not only mediates CD4^+^ T-cell activation but also responds to T-cell differentiation and Th responses.

Putative ASM inhibition has been used for the treatment of a variety of diseases in experimental models, for example, colitis,^29, 36^ and lung inflammation.^37^ In these *in vivo* and *in vitro* studies ASM mediated immune responses and heightened tissue damage. Blockade of ceramide production via pharmacological inhibition of ASM might attenuate macrophage activation and restore epithelium function during inflammation, and thus ameliorate disease injury in the tissues and organs.^29, 36, 37^ The present study further suggests pivotal roles of ASM in the regulation of adaptive immune responses, and identifies a potential target for the therapy of Th-dominant immune diseases.

In summary, we confirm that by interacting with both CD3 and CD28, ASM mediates CD3 and CD28 signals, and thereby controls CD4^+^ T-cell activation, as well as Th responses. ASM inhibition leads to decreased ceramide production, unresponsiveness of CD4^+^ T cell to CD3/CD28 engagements, and diminished Th responses.

## Materials and Methods

### Cells

Peripheral blood CD3^+^CD4^+^ T cells from healthy donors were isolated by Human CD4+ T cells Enrichment Cocktail Kit (STEMCELL Technologies, Vancouver, BC, Canada) with minor modification. Naive (CD3^+^CD4^+^CD45RA^+^) and memory (CD3^+^CD4^+^CD45RO^+^) CD4^+^ T cells were subsequently sorted by FACScan (BD Biosciences, San Jose, CA, USA). Cells were cultured in complete RPMI-1640 medium (Invitrogen, Carlsbad, CA, USA) supplemented with 2 mM l-glutamine, 100 U/ml penicillin, 100 *μ*g/ml streptomycin, 1% nonessential amino acids, and 10% FCS.

### Cell activation and proliferation assay

CD4^+^ T cells (1 × 10^6^/ml) were stimulated with anti-CD3/28 antibody-coated beads (the ratio of bead and cell is 1 : 1; Invitrogen) for 24 or 72 h. In some experiments, CD4^+^ T cells were pretreated with different inhibitors of ASM and during the experiment, including imipramine (0.2, 2, 10, and 20 *μ*M), desipramine (50 *μ*M), amitriptyline (5 *μ*M), and l-carnitine (1 mM). Cell activation was determined by flow cytometry of forward and side scatter, and labeling of cells with CD25 or CD69 antibodies. CFSE (Invitrogen) was used to determine cell proliferation. Cells were incubated with 2.5 *μ*M CFSE dye in PBS for 5 min at 37 °C. After four washes by complete culture medium, the cells were stimulated with anti-CD3/28 antibody-coated beads for 72 or 96 h. Cell recruitment and division were analyzed by labeling the cells with CFSE.

### Flow cytometry

For surface markers analysis, CD4^+^ T cells were stained in PBS containing 0.2% (wt/vol) BSA and the appropriate antibodies. For functional studies, CD4^+^ T cells were treated for 5 h with 50 ng/ml phorbol 12-myristate 13-acetate and 500 ng/ml ionomycin in the presence of 10 *μ*g/ml brefeldin A (Sigma-Aldrich, St. Louis, MO, USA). After surface staining, cells were permeabilized with saline buffer containing 0.5% saponin for 20 min, and subsequently incubated with fluorescein-conjugated antibodies. Flow cytometry data were acquired on a multi-color LSRII (BD Biosciences) and were analyzed with FlowJo software (Tree Star, Ashland, OR, USA).

### Immunoprecipitation and immunoblotting

For immunoprecipitation, cells were lysed on ice with RIPA buffer containing 1 mM EDTA, 50 mM Tris-HCl (pH 7.4), 150 mM NaCl, 1% NP-40, 0.25% sodium deoxycholate, and protease inhibitor mixture. The lysates were spun at 5000 × *g* for 5 min at 4 °C, and then the supernatant was taken and incubated with mouse anti-human ASM antibody (R&D Systems, Minneapolis, MN, USA) or mouse anti-human immunoglobulin G (IgG) (Santa Cruz Biotechnology, Paso Robles, CA, USA) for 3 h at 4 °C. After three washes, Protein G-Sepharose 4B (Sigma-Aldrich) was introduced according to product instruction. After washing, bound proteins were released from the immunoprecipitated complexes by adding 4 × SDS sample buffer (Bio-Rad, Hercules, CA, USA) and boiling for 5 min. After centrifugation, an equal volume of each sample was fractionated by SDS-PAGE.

For western blotting, 20 *μ*g of protein was loaded onto a Criterion XT Precast Gel (4–12% Bis-Tris; Bio-Rad) for separation by electrophoresis, and transferred to polyvinylidene difluoride membranes (Millipore, Billerica, MA, USA) using a semidry transfer system (Bio-Rad). After blocking, the membranes were incubated with primary antibodies, which included rabbit antibodies against phospho-Zap70 (Tyr319), phospho-PLC*γ*1 (Tyr783), phospho-ERK (Thr202/Tyr204), phospho-JNK (Thr183/Tyr185), phospho-PI3K p85 (Tyr458), phospho-Akt (Ser473), Akt, phospho-mTOR (Ser2448), mTOR, *β*-actin (Cell Signaling, Danvers, MA, USA), mouse against human CD3, and CD28 antibodies (BD Pharmingen, San Diego, CA, USA) respectively. After incubating with secondary antibodies, the immunoreactive bands were visualized using an enhanced chemiluminescence method (Thermo Scientific, Waltham, MA, USA). Mouse anti-*β*-actin antibody (Abcam, Cambridge, MA, USA) was used as an inner control. The relative expression levels of total and phosphorylated proteins were analyzed by Image J software (NIH, Bethesda, MD, USA) after normalization for the levels of *β*-actin and provided as a number under each band.

### ASM assay and TLC

Enzyme activity of ASM was measured as previously described with minor modifications.^38, 39^ Briefly, 1 × 10^6^ CD4^+^ T cells were stimulated with anti-CD3/28 beads (the ratio of bead and cell is 1 : 1; Invitrogen) for the indicated times. For single anti-CD3 or CD28 antibody stimulation, mouse anti-human CD3 or CD28 antibody (10 *μ*g/ml; BD Pharmingen) was mixed with crosslinking antibody (10 *μ*g/ml; BD Pharmingen) at 4 °C for 15 min, and then was introduced into CD4^+^ T cells. Stimulation was stopped by putting samples on dry ice for 10 s, and the cells were lysed in sodium acetate buffer (50 mM sodium acetate, pH 5.0, 0.5% NP-40).

Ceramide as the lipid cleavage product of ASM was then measured by TLC. Briefly, 10 *μ*g of cell lysates were incubated with sphingomyelin (0.2 mg, dissolved in a mixture of chloroform and methanol (1 : 1)) for 2 h and then loaded onto a TLC plate, followed by chromatographic separation using a solvent system of chloroform and methanol (3 : 2). Ceramide production was then visualized in iodine vapor, identified using a standard run-in parallel, and scanned by a scanner. Relative ceramide levels were evaluated by Image J software (NIH).

### Th differentiation

Sorted CD4^+^ T cells were *in vitro* cultured in RPMI-1640 supplemented with 10% (vol/vol) FCS and antibiotics. A total of 1 × 10^6^/ml CD4^+^ T cells were stimulated with anti-CD3/28 antibody-coated beads (the ratio of bead and cell is 1 : 1; Invitrogen) for 3 or 4 days. For pharmacological inhibitor treatments, naive CD4^+^ T cells were incubated with different inhibitors of ASM at the onset of cultures. For Th1 cell differentiation, 10 ng/ml IL-12 and 10 *μ*g/ml anti-IL-4 antibody were added to the culture media. For Th17 condition, 50 ng/ml IL-6, 30 ng/ml IL-1*β*, 30 ng/ml IL-23, and 2 ng/ml TGF-*β* were included in cultures. For Th2 condition, 30 ng/ml IL-4 and 10 *μ*g/ml anti-IFN*γ* antibody were added. All cytokines and antibodies were purchased from R&D Systems.

### Small hairpin RNA

Healthy CD4^+^ T cells were infected separately with an empty shRNA vector control (sh-C, pLKO.1-puro) or different human ASM shRNA (sh-1: NM_000543.2-367s1c1, TRCN0000049014; sh-2: NM_000543.2-1467s1c1, TRCN0000049015) lentiviral transduction particles (Invitrogen), according to the manufacturer's instructions. Recombinant lentiviral particles were produced by transient transfection of 293FT cells as described.^40^ CD4^+^ T cells were infected with lentiviral particles according to previously described method with a minor modification.^41^ Briefly, after stimulation with anti-CD3/28 antibodies (both 2 *μ*g/ml) and human recombinant IL-2 (R&D Systems) (1 ng/ml) for 24 h, CD4^+^ T cells were infected by lentiviral particles by centrifugation at 2300 r.p.m. for 60 min at room temperature in the presence of polybrene (8 *μ*g/ml). These cells were replaced with fresh media containing human recombinant IL-2 (R&D Systems) (1 ng/ml) for additional 48 h, and then selected with puromycin (2.25 *μ*g/ml) for 5–10 days. The surviving cells were selected using a Dead Cell Removal Kit (Miltenyi Biotec, San Diego, CA, USA).

### Confocal microscopy

Cells were double stained with antibodies to CD3 (APC-CD3; BioLegend, San Diego, CA, USA) and CD28 (FITC-CD28; BioLegend), and visualized under a confocal microscope (Zeiss LSM 510 Meta; Zeiss, Peabody, MA, USA). For intracellular staining, the cells were permeabilized with 0.2% Triton X-100 for 3 min, and incubated with primary anti-ASM antibody (Cell Signaling) and secondary PE-Cy7-conjugated antibody (Santa Cruz Biotechnology) sequentially.

### Statistics

Differences between experimental groups were assessed by one-way analysis of variance, the Tukey–Kramer multiple comparison test (for multiple groups), or Student's *t-*test (for comparisons between two groups). *P<*0.05 was considered to be statistically significant.

## Abbreviations

ASM: acid sphingomyelinase
CFSE: carboxyfluorescein diacetate succinimidyl ester
IL: interleukin
JNK: c-Jun N-terminal kinase
MAPK: mitogen-activated protein kinase
mTOR: mammalian target of rapamycin
PI3K: phosphoinositide 3-kinase
PLC-*γ*1: phospholipase C-*γ*1
shRNA: small hairpin RNA
TCR: T-cell receptor
Th: T-helper cell
TLC: thin-layer chromatography
